# Comparative Genomics of a Bovine *Mycobacterium tuberculosis* Isolate and Other Strains Reveals Its Potential Mechanism of Bovine Adaptation

**DOI:** 10.3389/fmicb.2017.02500

**Published:** 2017-12-12

**Authors:** Xuekai Xiong, Rui Wang, Dachuan Deng, Yingyu Chen, Han Liu, Tianqi Wang, Jieru Wang, Xiaojie Zhu, Xifang Zhu, Yongqiang Zhu, Xinyan Lu, Huanchun Chen, Huajun Zheng, Aizhen Guo

**Affiliations:** ^1^National Key Laboratory of Agricultural Microbiology, Huazhong Agricultural University, Wuhan, China; ^2^College of Veterinary Medicine, Huazhong Agricultural University, Wuhan, China; ^3^Shanghai-MOST Key Laboratory of Health and Disease Genomics, Chinese National Human Genome Center at Shanghai, Shanghai, China

**Keywords:** *Mycobacterium tuberculosis*, genome, sequencing, cattle, zoonosis, tuberculosis

## Abstract

The *Mycobacterium tuberculosis* complex causes tuberculosis (TB) in humans and other animal species, but *Mycobacterium tuberculosis* has a distinct host preference to humans. The present study aimed to determine whether a bovine *M. tb* strain 1458 has evolved some genetic properties in their genome that might be associated with their bovine adaptation. The genome of the *M. tb* strain 1458 was sequenced and subjected to an extensive comparative genomic analysis. A phylogenetic analysis showed that strain 1458 is most closely related to a Chinese *M. tb* strain, CCDC5079, of the same Beijing family. Compared with three human *M. tb* Beijing family strains, the strain 1458 has the fewest unique genes. However, there are most (21) IS*6110* insertion sequences in the strain 1458 genome at either intragenic or intergenic sites, resulting in the interruption of 11 genes including three PPE family-encoding genes (PPE16, PPE38, and PPE59). Only the strain 1458 genome has the upstream insertion in *esxS* and *phoP* genes. PCR confirmed four upstream insertions and qPCR determined that transcription of *esxS, phoP, dnaN*, and *ctpD* genes differed significantly between *M. tb* strain 1458 and H37Rv or *M. bovis*. A Kyoto Encyclopedia of Genes and Genomes pathway enrichment analysis revealed that the genes affected by non-synonymous SNPs are enriched in RNA polymerase. Moreover, 127 of the 133 unique SNPs in strain 1458 are either different to those in the *M. bovis* genome. In conclusion, some critical genes responsible for bacterial virulence and immunogenicity were interrupted in the genome of bovine *M. tb* strain 1458 by IS insertions and non-synonymous SNPs, which might contribute to its bovine adaptation, and the modification of its virulence and immunogenicity in cattle.

## Introduction

Tuberculosis (TB) is one of the most serious infectious diseases of humans worldwide, in terms of the numbers of cases and deaths. According to the Global Tuberculosis Report 2016 from the World Health Organization, there were 10.4 million new TB cases and 1.4 million TB deaths in 2015 (World Health Organization, 2016). Although TB is known to be a zoonosis, the pathogenic members of the *Mycobacterium tuberculosis* complex (MTBC) have certain host preferences. For instance, human TB is usually caused by *Mycobacterium tuberculosis*, while bovine TB is caused by *Mycobacterium bovis*. Because direct evidence of bovines infected by *M. tb* is limited, *M. tb* is usually thought to be avirulent to cattle, or only cause transient infections in cattle (Whelan et al., 2010). In contrast, in addition to cattle, *M. bovis* has the capacity to cause TB in a broad of mammal hosts, such as humans, deer, badgers, and possums (Perez-Lago et al., 2014). In humans, it was estimated that ~10% of TB cases are caused by *M. bovis* which might vary with the regions, although it is thought that *M. bovis* cannot be transmitted among humans (Berg and Smith, 2014).

However, recently, many studies have reported the isolation of *M. tb* in cattle herds, with prevalence of 4.7–30.8% in African and Asian countries, where there are ~80% of the global human TB patients (Prasad et al., 2005; Chen et al., 2009; Milian-Suazo et al., 2010; Fetene et al., 2011). In addition, some other evidence indicates that *M. tb* strains can infect bovines for a long period, rather than causing transient infections, and that these strains can be transmitted among cattle and cause immunological and pathological responses (Chen et al., 2009, 2013). However, the overall risk that *M. tb* infects bovines, thereby threatening both cattle and human health, needs to be evaluated further.

China is one of 22 high-burden TB countries. The dominant *M. tb* genotype (accounting for ~80% of human TB cases) belongs to the Beijing family in China (Han et al., 2007). In our previous study, we isolated some Beijing family *M. tb* strains from cattle diagnosed as bovine TB (Chen et al., 2009), and a cattle experiment with one representative strain 1458, demonstrated that this bovine *M. tb* strain could cause immune responses and metabolomics disorders in cattle, just like those caused by *M. tb* strain H37Rv and *M. bovis* strain AF2122_97 (Chen et al., 2013). The present study aimed to determine whether this strain 1458 has evolved some genetic characteristics that might contribute to its successful infection of cattle. The full genome of *M. tb* strain 1458 was sequenced, and a comprehensive comparative analysis of the genomes was performed of strain 1458, other *M. tb* strains, including other Beijing family *M. tb* isolates from humans, and a *M. bovis* type strain.

## Materials and methods

### Bacterial growth and DNA/RNA extraction

Bovine *M. tb* strain 1458 was isolated in this lab. The *M. tb* strain H37Rv and *M. bovis* strain AF2122_97 were kindly offered by Prof. Chuanyou Li from Beijing Tuberculosis and Thoracic Tumor Research Institute. The strains were cultured to mid-log phase in 20 ml of Middle brook 7H9 medium (Becton Dickinson and Company, Franklin Lakes, NJ, USA) that was supplemented with 10% oleic acid, albumin, dextrose, and catalase medium (Becton Dickinson and Company) and 0.05% Tween 80 (Amresco Inc., Solon, OH, USA), with agitation at 37°C for 1–2 weeks in a biosafety level 3 facility at Huazhong Agricultural University (Wuhan, China). The culture of *M. tb* strain 1458 was centrifuged at 10,000 × g and 4°C for 10 min, and DNA was extracted using cetyl trimethylammonium bromide, and 2 μg of DNA was used for sequencing. The total RNA of all the three strains was extracted using FastRNA Blue Kit (MP Biomedicals, Shanghai, China), and was reverse-transcribed into cDNA using a HiScript II One Step RT-PCR Kit (Vazyme Biotech, Nanjing, China).

### High-density pyrosequencing and sequence assembly of the genome

Complete genomic sequencing was conducted using a Roche GS FLX system (454 Life Sciences Corp, Branford, CT, USA; Margulies et al., 2005). A total of 267,232 reads amounting 128 Mb of raw data (average read length of 478 bp), were obtained, resulting in 29-fold genome coverage. Assembly was performed using the GS de novo Assembler software from SFF file with default parameters, which will remove reads shorter than 50 bp. A total of 113 contigs ranging from 500 to 203,016 bp were produced. The relationships among the contigs were determined by multiplex polymerase chain reaction (PCR) (Tettelin et al., 1999). Then, gaps were filled in by sequencing the PCR products using ABI 3730XL capillary sequencers (Applied Biosystems, Waltham, MA, USA). Phred, Phrap, and Consed software packages (http://www.phrap.org/phredphrapconsed.html) were used for final assembly and editing, and the low-quality regions (the bases with phred quality score <20) of the genome were re-sequenced. The final sequencing accuracy was 99.99%.

### Genome annotation

Putative coding sequences were identified by Glimmer 3.02 (Delcher et al., 1999), and the peptides shorter than 30 amino acids were eliminated. Insertion sequences were first detected using the IS Finder database (https://www-is.biotoul.fr/) by the default parameters. The tRNA genes were predicted by tRNAScan-SE (Lowe and Eddy, 1997), and rRNA genes were predicted by RNAmmer (Lagesen et al., 2007) using the default parameters. Functional annotation of the coding sequences was performed by searching against NCBI non-redundant protein database using the protein Basic Local Alignment Search Tool (BLASTP) (E-value is set to 1e-10) (Altschul et al., 1997) and the Conserved Domain Database (CDD) (Marchler-Bauer et al., 2007) by reversed positon specific BLAST with E-value < 1e-5. The pseudogenes were identified by BLASTP, with an amino acid alignment length <20% of the referenced proteins' amino acid lengths. Metabolic pathways were constructed using the Kyoto Encyclopedia of Genes and Genomes (KEGG) database (Kanehisa et al., 2004). Genome comparisons were performed using Mauve (Darling et al., 2004). The genome atlas was drawn using GenomeViz1.1 (Ghai et al., 2004). A new pan-genome analysis pipeline (Zhao et al., 2012) was used to identify the orthologs among the three genomes, with the MP method using the following settings: coverage >50% and identity >50%.

### Phylogenetic tree construction

Orthologs of known *Mycobacterium tuberculosis complex* (MTBC) genomes were obtained from the National Center for Biotechnology Information database (https://www.ncbi.nlm.nih.gov/genome/). The phylogenetic position of the genome of *M. tb* strain 1458 was determined based on orthologous proteins. Concatenated protein sequences of orthologous *Mycobacterium* species proteins were first aligned using MAFFT (Katoh and Standley, 2013), and then the conserved alignment blocks were extracted by the MEGA6 program (Tamura et al., 2013). A maximum likelihood tree was built with PHYML (Guindon et al., 2010) with 1,000 bootstrap replications and the following parameters: “JTT” for the substitution model; “estimated” for the proportion of invariable sites; “estimated” for the gamma distribution parameters; “4” for the number of substitution categories; “yes” to the optimized tree topology; and “BIONJ” for the starting tree.

### PCR amplification primers and conditions

PCR was used to confirm IS*6110* insertion in the upstream of the genes *esxS, phoP, dnaN*, and ctpD in *M. tb* strain 1458. Primer sequences and products' size were listed in Table 1, and the PCR conditions were as follows: 95°C for 5 min, followed by 35 cycles of 95°C for 60s, 62°C for 60s, and 72°C for 90s, and ended with a final extension at 72°C for 10 min.

**Table 1:** The PCR primer sequences in this study.

**Genes**		**Primer sequences (5′ → 3′)**	**Products' size**
esxS	F	GGTGCCAGACATCGACTGAT	1,784/429
	R	CAGGCGTTTCATCAGGGAGA	
phoP	F	GCATCACCCAACGCTTGTTT	1,740/387
	R	ACCGACAGCAGTTCAACGAT	
dnaN	F	CCAGTCACGACAGATTGCGA	1,739/386
	R	TGACTGTGGGGTGTGTGTTG	
ctpD	F	GTTCCTGCGTCCCTACACTC	1,702/349
	R	CATCAAGTGCCTTGTTCCGC	

### Absolute quantitative real-time PCR standards

The absolute quantitative real-time PCR (qPCR) was used to determine the copy number of four typical genes (*esxS, phoP, dnaN*, and *ctpD*). The qPCR standards for these genes were firstly prepared. The primer sequences and products' size were listed in Table 2, and the four gene fragments were amplified and sequences verified. Then, the four PCR products were cloned into the pMD18-T vector (TaKaRa, Dalian, China) to construct the four recombinant plasmids, pMD-phoP, pMD-esxS, pMD-dnaN, and pMD-ctpD. The concentrations of the four recombinant plasmids were measured with a NanoDrop 2000 spectrophotometer (Thermo Scientific, Waltham, MA, USA), and the copy number was calculated as the following formula: number of copies = [recombinant plasmid concentration (ng) × 6.022 × 10^23^]/[recombinant plasmid length × 1 × 10^9^ × 650].

**Table 2:** The qPCR primer sequences in this study.

**Genes**		**Primer sequences (5′ → 3′)**	**Products' size**
esxS	F	TGTTGGATGCCCATATTCC	106
	R	GACATCGCCTGCTGCTC	
phoP	F	CGGCGTTGTTCCTGAC	154
	R	TCCTTGTTGCCCTTGC	
dnaN	F	TGTCGTGGGTGGCTAAA	162
	R	AAACGCTTCCAGGAGAAA	
ctpD	F	TGAACGGATCGGGTGTATT	154
	R	ATGCCCAGGGAGTAGCG	

The absolute qPCR was performed by using AceQ qPCR SYBR® Green Master Mix (Vazyme Biotech, Nanjing, China). The volume of each reaction was 10 μl, including 1.5 μl of cDNA, 0.25 μl of each primer, 5 μl of mixture and double distilled water to a volume of 10 μL. The whole experiment was programmed in an ABI ViiA™ 7 Real-Time PCR System (Applied Biosystems, Waltham, MA, USA) as follows: 95°C for 10 min for the hot-start, followed by 40 cycles of 95°C for 30 s, 58°C for 30 s, and 72°C for 30 s, and a melting curve program. The fluorescence signal was collected at the end of each elongation step. Ten-fold serial dilutions of the four recombinant plasmids DNA from 10^1^ to 10^10^ copies/μl were prepared to generate a standard curve for absolute qPCR. The results are expressed as the mean ± the standard deviation (SD) of the mean. Differences in the data between the groups were analyzed by ANOVA using the GraphPad Prism5 and *p*-values < 0.05 and < 0.01 were considered to be statistically significant (^*^) and very significant (^**^).

## Results

### Genome features of *M. tb* strain 1458

**Figure 1:**
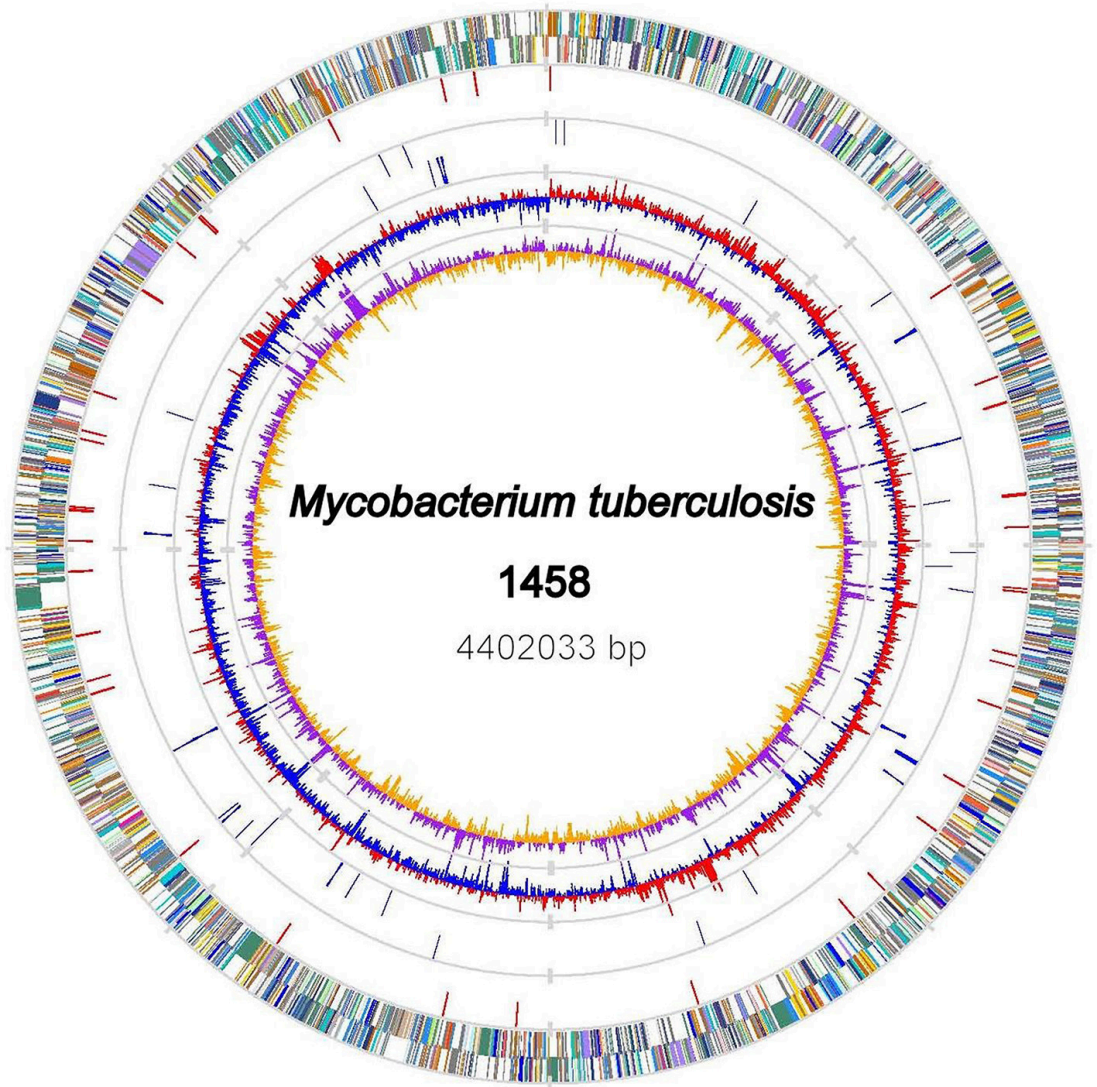
Chromosome Atlas of *M. tb* 1458. The scale is shown by the outer black circle. Moving inside, the 1st and 2nd circles illustrate predicted coding sequences on the plus and minus strands respectively, colored according to different functional categories. The 3rd circle displays IS*6110* elements (red). The 4th circles tRNAs (blue) and ribosomal RNA genes (gray). The 5th and 6th (innermost) circles represent mean centered G+C content of the genome (red-above mean, blue-below mean) and GC skew (G−C)/(G+C), respectively, calculated by using a 1-kb window in steps of 500 bp.

**Figure 2:**
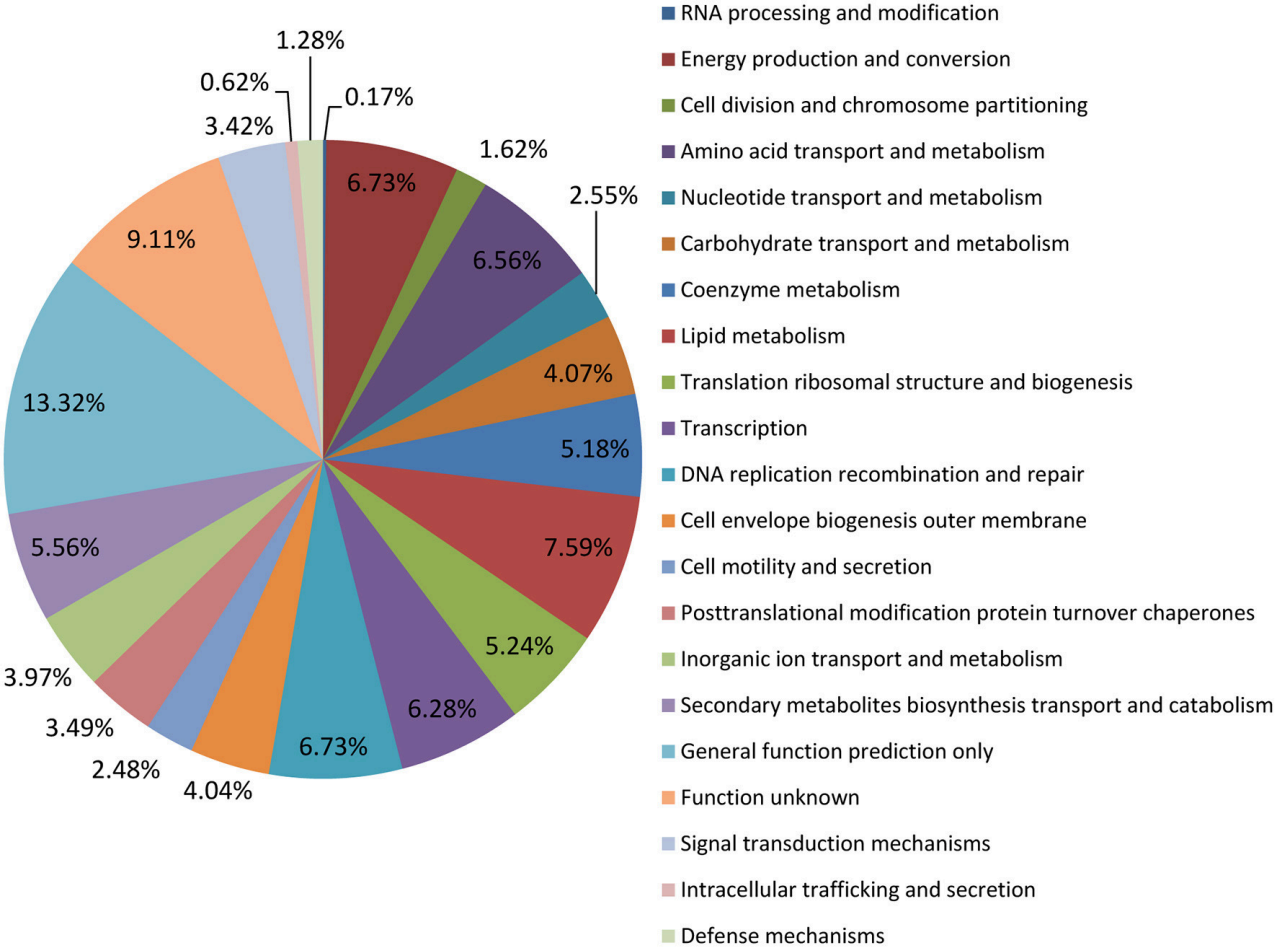
COG annotation *M. tb* 1458 genome.

The genome of the *M. tb* 1458 strain contains a single circular chromosome of 4,402,033 bp with a GC content of 65.6% (GenBank accession no: CP013475; Figure 1). We identified 4,349 open reading frames (ORFs) in the genome, with an average length of 915 bp, which occupy 90.4% of the genome. Among these ORFs, 2,898 (66.6%) genes could be classified into Clusters of Orthologous Groups (COGs) families comprising 21 functional categories (Figure 2, Table S1).

**Figure 3:**
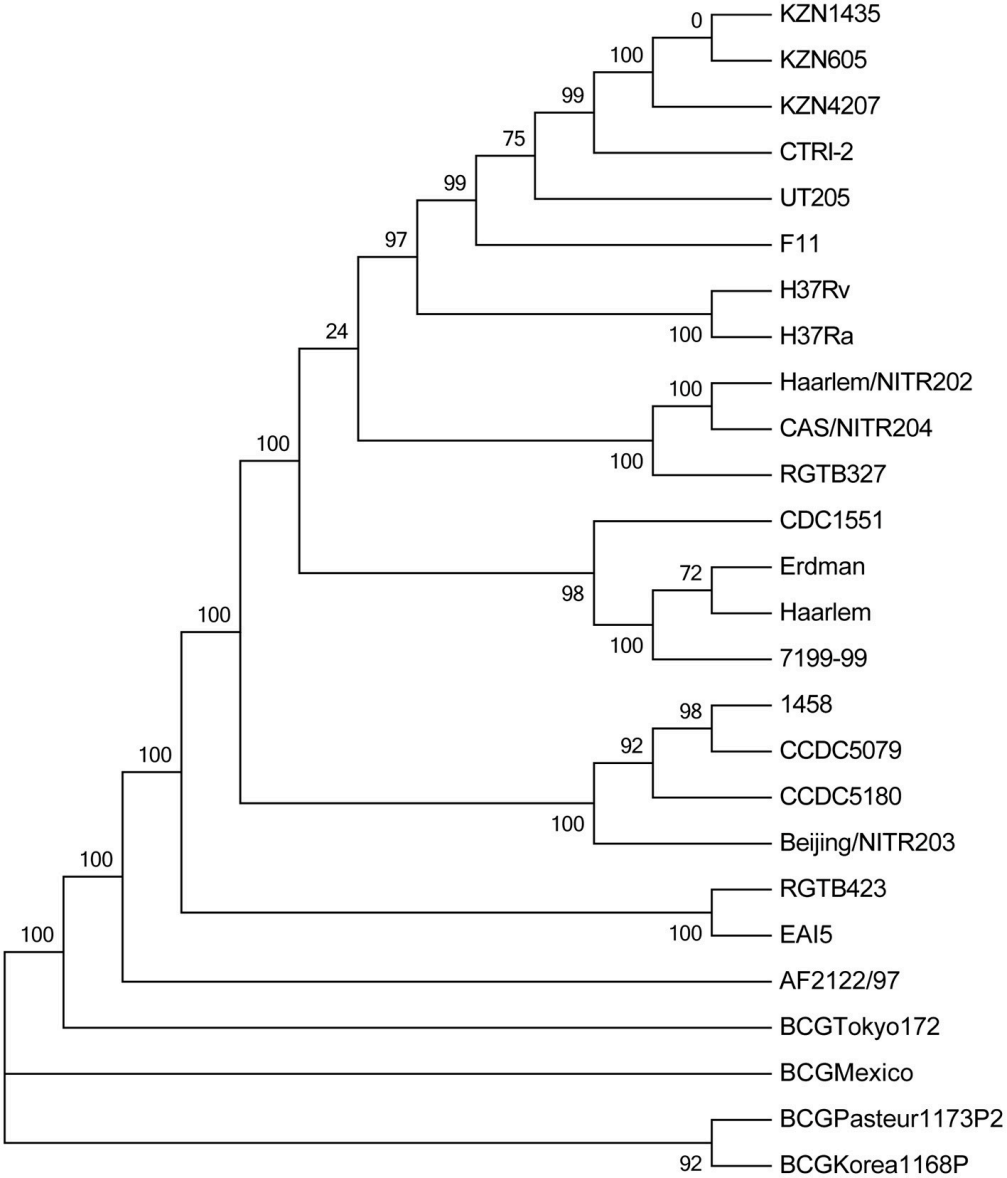
Phylogenetic tree of sequenced members of *Mycobacterium tuberculosis* complex (MTBC).

To perform a comprehensive comparative genomic analysis, a total of 25 mycobacterial genomes in addition to that of *M. tb* strain 1458 were retrieved from the GenBank database, and a phylogenetic analysis was performed. As expected, all four Beijing strains fell within the same cluster, and the 1458 strain was most closely related to the Beijing family Chinese strain CCDC5079 (Figure 3).

### Intragenic and intergenic is insertions

In total, 18 kinds of complete IS elements were found in the *M. tb* 1458 genome using online tools (https://www-is.biotoul.fr/). Basically, the IS elements belong to the IS*256*, IS*607*, IS*3*, and IS*521* families (Table S2). In addition, we identified 21 copies of IS*6110* in strain 1458, compared with 16 copies in strain H37Rv. Among them, 11 IS*6110*s are intragenic insertions (Table 3), and the disrupted ORFs include three PPE family genes (PPE16, PPE38, and PPE59), and two genes that encode the cell wall proteins Mmpl12 and LprP. PPE family proteins have been well demonstrated to play a role in the antigenic variation of *M. tb* strains in humans (Dheenadhayalan et al., 2006), and, therefore, the disruption of these genes might affect the membrane structure and alter the antigen profile of strain 1458 in cattle, thereby probably allowing *M. tb* to evade the host immune system, facilitating an ongoing infection, and further bacterial transmission. Moreover, seven of these 11 intragenic insertions generated 5-, 4-, 4-, 6-, 3-, 3-, and 4-bp direct repeat sequences next to the IS*6110* insertion sites (Table 3).

**Table 3:** Eleven intragenic IS*6110*s distributed within ORFs in *M. tb* 1458.

**Location (nt)**	**Locus**	**Gene or product**	**DR**
1070104–1071458	BTB1458_1064, BTB1458_1067	lprp	CAGGC
1259595–1260949	BTB1458_1273	PPE16	AAGC
1542095–1543449	BTB1458_1535, BTB1458_1538	Desaturase-related protein	GAGG
1716764–1718118	BTB1458_1711	mmpl12	–
2247431–2248785	BTB1458_2235, BTB1458_2238	Hypothetical protein	TCAAGG
2621110–2622464	BTB1458_2606, BTB1458_2609	PPE38	–
3102221–3103575	BTB1458_3111	Hypothetical protein	–
3354543–3355897	BTB1458_3335	Hypothetical protein	GGC
3472672–3474026	BTB1458_3459	Hypothetical protein	CGA
3781730–3783084	BTB1458_3751, BTB1458_3754	idsB	AATC
3834127–3835481	BTB1458_1084	PPE59	–

In accordance to previous studies that determined that intergenic regions are preferential targets for IS*6110* insertions, we found that the other 10 IS*6110*s are intergenic insertions at 10 different sites (Table 4), and that they are located upstream of genes such as *phoP, esxS, dnaN, ctpD*, the PE22-encoding gene, four hypothetical protein-encoding genes, and one alanine-rich protein-encoding gene. These genes encode proteins that play significant roles in *M. tb* infection and the development of the host immune response. For example, *phoP* encodes one component of the PhoP-PhoR two-component signaling system, which orchestrates a complex transcriptional program that is essential for mycobacterial growth and virulence (Soto et al., 2004); *esxS* encodes a member of the 6-kDa early secretory antigenic target (ESAT-6) family of proteins, which are immunogenic and protective (Meher et al., 2006); *dnaN* encodes a key component of DNA polymerase III, which is a protein essential for many important DNA transactions including replication and repair (Machaba et al., 2016); *ctpD* encodes a member of the Co^2+^/Ni^2+^-transporting P_1B4_-ATPase sub-group, which is important for Co^2+^ and Ni^2+^ homeostasis (Raimunda et al., 2014) and PE22 belongs to the PE/PPE family, which plays an important role in antigenic variation, as mentioned above (Dheenadhayalan et al., 2006). In this study, the 10 intergenic IS*6110* insertions are located in the upstream regions of genes, four of which are in the same orientation (5′ to 3′) as the associated genes, while the other six had the reverse orientation. In addition, six of the 10 intergenic insertions generated direct repeat sequences of 3-, 4-, 3-, 4-, 5-, and 3-bp (Table 4).

**Table 4:** Ten intergenic IS*6110*s distributed between ORFs in *M. tb* 1458.

**Location (nt)**	**Orientation 5′–3′**	**Locus**	**Gene or product**	**Distance**	**DR**
1595–2949	+	BTB1458_0004	dnaN	460 bp upstream	ATT
845802–847156	–	BTB1458_0836	phoP	132 bp upstream	GTAT
884675–886029	+	BTB1458_0879	Hypothetical protein	39 bp upstream	CCA
1656829–1658183	–	BTB1458_1647	ctpD	61 bp upstream	CGTT
1977846–1979200	–	BTB1458_1950	Hypothetical protein	313 bp upstream	–
2355774–2357128	+	BTB1458_2341	PE22	539 bp upstream	CACAT
3356375–3357729	+	BTB1458_3339	esxS	11 bp upstream	CAG
3531885–3533239	–	BTB1458_3520	Alanine rich protein	57 bp upstream	–
3695205–3696559	–	BTB1458_3692	Hypothetical protein	242 bp upstream	–
3830213–3831567	–	BTB1458_3805	Conserved hypothetical protein	172 bp upstream	–

**Figure 4:**
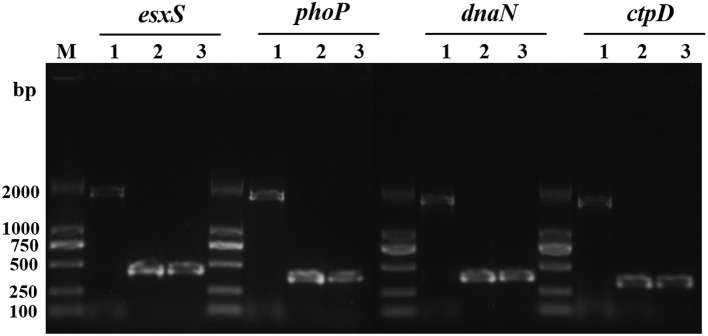
IS*6110* insertion in the upstream of *esxS, phoP, dnaN*, and *ctpD* genes of *M. tb* 1458 was confirmed with PCR. Lane 1, *M. tb* strain 1458; lane 2, H37Rv; lane 3, *M. bovis* AF2122_97.

It was previously demonstrated that IS*6110* can act as a promoter when it is inserted in the upstream region of a gene near the transcriptional start site. Firstly, with PCR, we verified IS*6110* insertions in the regions of four typical genes. As expected, the amplification products including IS*6110* upstream insertion and the genes *esxS, phoP, dnaN*, and *ctpD* were 1,784, 1,740, 1,739, and 1,702 bp, respectively. In contrast, for both *M. tb* H37Rv and *M. bovis* AF2122_97, because IS6110s were absent in the upstream of these four genes, PCR products are in size of 429, 387, 386, and 349 bp correspondingly (Figure 4).

**Figure 5:**
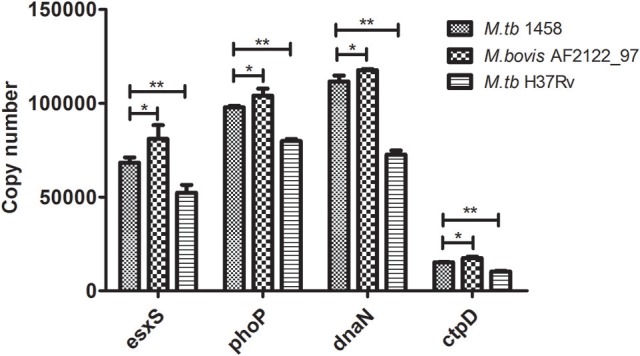
The copy numbers of *esxS, phoP, dnaN*, and *ctpD* transcripts in *M. tb* 1458, H37Rv and *M. bovis* AF2122_97. The copy numbers of four typical genes (*esxS, phoP, dnaN*, and *ctpD*) in *M. tb* strain 1458, H37Rv and *M. bovis* AF2122_97 were determined by absolute quantitative real-time PCR. The data shown are the mean ± SD of three replicates. ^*^ represents a significant difference (*p* < 0.05), and ^**^ a very significant difference (*p* < 0.01).

Then, the copy numbers of *esxS, phoP, dnaN*, and *ctpD* transcripts were determined by absolute qPCR. First of all, the transcription of all four genes in *M. tb* 1458 was significantly higher than *M. tb* H37Rv (*p* < 0.01), although the absolute copies of transcripts of the four genes are different; and when the four gene transcription of these three strains was compared, the *M. bovis* strain expressed significantly highest activity, *M. tb* 1458 followed, and *M. tb* H37Rv demonstrated the least activity (Figure 5).

### Global comparison of the gene repertoires of *M. tb* strains 1458, H37Rv, and *M. bovis* strain AF2122_97

**Figure 6:**
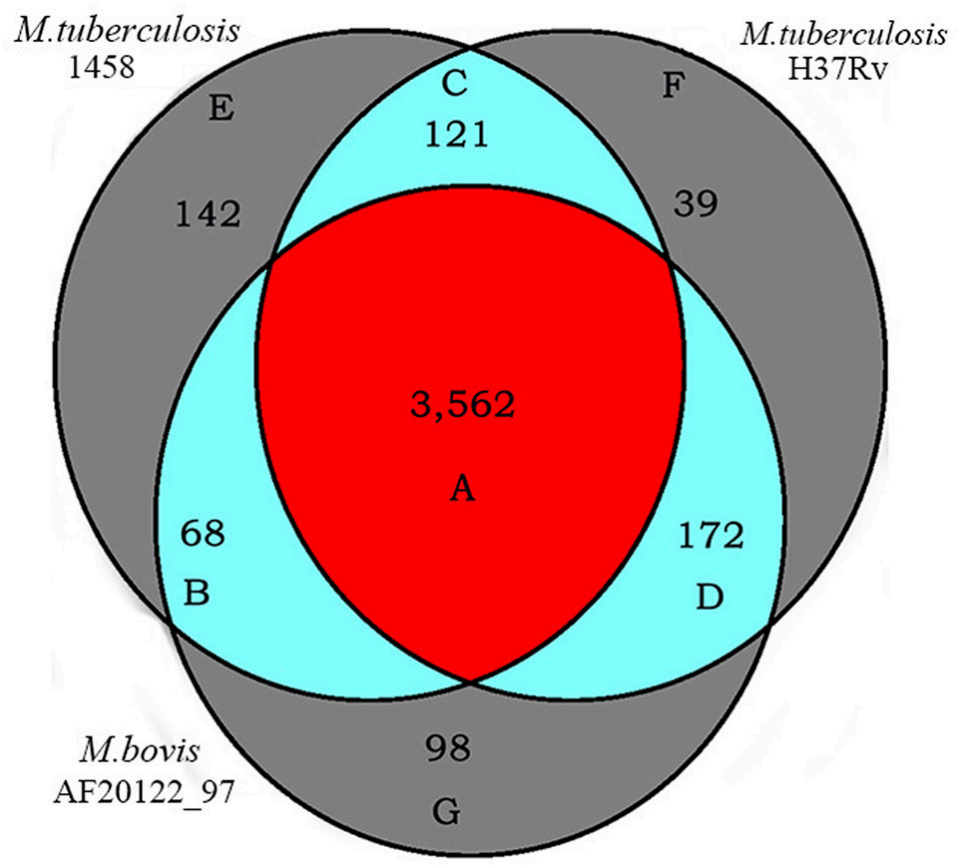
Orthologs of *M. tb* 1458, H37Rv and *M. bovis* AF2122_97. Numbers of orthologous genes of *M. tb* 1458, H37Rv and *M. bovis* AF2122_97 genomes were evaluated using the PGAP (http://pgap.sf.net). The core genome of these three strains consists of 3,562 genes, as *M. tb* 1458, H37Rv and *M. bovis* AF2122_97 common orthologs. Letters refer to Table S3 giving the list of orthologs.

A search for orthologous genes in the genomes of *M. tb* strain 1458 (GenBank accession no. CP013475), *M. tb* strain H37Rv (GenBank accession no. NC_000962), and *M. bovis* strain AF2122_97 (GenBank accession no. NC_002945) identified various clusters of orthologs that were specific to each genome or shared by two or three of the genomes (Figure 6, Table S3).

A total of 3,562 orthologs were identified as the core genome of the three genomes (Figure 6, Table S3-1). These orthologous genes account for 82.8% of the total genes of the three genomes (identified as the pan genome). Among them, 2,818 orthologous genes belong to the COGs classes that are essential for mycobacterial survival and nutrition utilization, including the categories of translation, ribosomal structure, and biogenesis, DNA replication, recombination and repair systems, and carbohydrate, amino acid, nucleotide, and inorganic ion transport and metabolism systems, as well as transcription and energy production and conversion systems. In contrast, 744 of the orthologous genes did not belong to any COGs classes, and they included genes encoding 322 hypothetical proteins, 172 virulence-related factors, 58 transmembrane proteins, 51 membrane proteins, 21 integral membrane proteins, and 10 transcriptional regulatory proteins. The virulence-related factors encoded by the core genome include 127 PE/PE-polymorphic GC-rich repetitive sequence (PGRS)/PPE family proteins, 65 lipoproteins, 47 antitoxins, 42 exported/secreted proteins, 32 epithelial-restricted with serine box (ESX) secretion system proteins (Meher et al., 2006), 23 mammalian cell entry (MCE)-associated proteins (Zhang et al., 2012), 13 ESAT-6 like proteins, 11 toxins (Ramage et al., 2009), four low-molecular-weight antigens, two immunogenic proteins, one integration host factor, and other related factors.

Unique genes were also identified in the three genomes (Figure 6, Tables S3-2–S3-4). One hundred and forty-two genes were considered to be specific for *M. tb* strain 1458 (Figure 6, Table S3-2). Among them, 70% (100/142) genes were identified as encoding hypothetical proteins, including 37 truncated genes and 63 hypothetical protein-encoding that are unique to the strain 1458 genome, compared with the other two genomes. In addition to these hypothetical protein-encoding, 26 other functional genes were also identified as being specific to the strain 1458 genome, including 13 PE-PGRS/PPE family-encoding genes, eight transposase-encoding genes, and one putative ESAT-6 like protein-encoding gene, *esxL* (BTB1458_4012). The remaining 16 specific genes are truncated genes, accounting for 37.3% of the specific genes. Most of the truncated genes have orthologs in the other two genomes (Figure 6, Tables S3-5–S3-7). Thirty-nine genes were considered to be specific for *M. tb* strain H37Rv (Figure 6, Table S3-3), including 14 genes that encode hypothetical proteins, 11 PE-PGRS/PPE family protein-encoding genes, four antitoxin-encoding genes, and two ESAT-6-like protein-encoding genes. Ninety-eight genes were considered to be specific for *M. bovis* strain AF2122_97 (Figure 6, Table S3-4), including 51 genes that encode hypothetical proteins, six PE/PE-PGRS/PPE family protein-encoding genes, four transmembrane transport protein-encoding genes, four transposase-encoding genes, and one lipoprotein-encoding gene, *lppOa*.

Overall, the specific hypothetical proteins and PE-PGRS/PPE family proteins seem to be more responsible for the different virulence characteristics of the three strains. Because half of the genes encoding the hypothetical proteins and most of the genes encoding the PE-PGRS/PPE family proteins are orthologous, the differences at the protein level may be attributed to frameshift mutations in these genes. This coincides with the highly similar structures of these three genomes (Figure S1).

### Potential effect of SNPs on the *M. tb* 1458 genome

A search for SNPs between the *M. tb* 1458 and H37Rv genomes identified 2,439 SNPs, including 1,400 non-synonymous SNPs, 838 synonymous SNPs, and 201 intergenic SNPs (Table S4-1). The non-synonymous SNPs affected 715 genes in *M. tb* strain H37Rv. However, the comparison between the *M. tb* 1458 and *M. bovis* AF2122_97 genomes identified more SNPs (2,958), including 1636 non-synonymous SNPs, 980 synonymous SNPs, and 342 intergenic SNPs. The non-synonymous SNPs affected 1,037 genes in *M. bovis* strain AF2122_97 (Table S4-2).

Because the 181 non-synonymous SNP-affected genes in *M. tb* strain H37Rv and the 246 non-synonymous SNP-affected genes in *M. bovis* strain AF2122_97 were involved in different pathways, a KEGG pathway enrichment analysis was performed, and it revealed significant enrichment of these genes in some pathways (Tables 5, 6). In the *M. tb* H37Rv genome, the non-synonymous SNP-affected genes were enriched in carbohydrate metabolism, biosynthesis of other secondary metabolites, and replication and repair (Table 5), while in the *M. bovis* AF2122_97 genome, the genes were enriched in carbohydrate metabolism and transcription (Table 6). Among them, there is one non-synonymous SNP-affected gene (*aceAa*) (Rv1915) that encodes an isocitrate lyase that is specific for the *M. tb* H37Rv genome. It is known that isocitrate lyase mediates the broad antibiotic tolerance of *M. tb* (Nandakumar et al., 2014), and two isocitrate lyase genes, namely *aceAa* and *aceAb*, were found to commonly exist in these three genomes. However, the *aceAa* gene, which has a single-base-pair overlap resulting in two ORFs (*aceAa/b*), is specific for the *M. tb* H37Rv genome. However, it is read as one continuous ORF in *M. bovis* strain AF2122_97 and *M. tb* strain 1458, as well as other mycobacterial strains (Zu Bentrup et al., 1999; Nandakumar et al., 2014; Shukla et al., 2015). We speculate that the disruption of *aceA* in *M. tb* strain H37Rv may be the result of long-term laboratory growth in which lipids were not the main carbon source, while the redundancy in isocitrate lyase may provide a greater chance for bacterial survival in host cell debris where lipids are used as a carbon source, indicating that the *aceA* gene may be important in host selection (Zu Bentrup et al., 1999; Shukla et al., 2015).

**Table 5:** The KEGG pathways enriched with non-synonymous SNPs affected genes in *M. tb* H37Rv genome.

**Pathways**	**A**	**B**	**C**	**D**	***P*-value**	***Q*-value**
Carbohydrate metabolism	48	210	181	1,084	0.00	0.06
Biosynthesis of other secondary metabolites	6	17	181	1,084	0.01	0.12
Replication and repair	13	52	181	1,084	0.04	0.21
Transcription	5	17	181	1,084	0.05	0.21
Signal transduction	12	53	181	1,084	0.09	0.30
Membrane transport	15	72	181	1,084	0.13	0.37
Glycan biosynthesis and metabolism	4	19	181	1,084	0.20	0.46
Metabolism of terpenoids and polyketides	25	134	181	1,084	0.22	0.46
Metabolism of other amino acids	14	80	181	1,084	0.35	0.66
Lipid metabolism	17	100	181	1,084	0.40	0.68
Nucleotide metabolism	14	85	181	1,084	0.45	0.70
Amino acid metabolism	41	259	181	1,084	0.63	0.79
Energy metabolism	20	135	181	1,084	0.69	0.79
Folding, sorting and degradation	6	47	181	1,084	0.69	0.79
Xenobiotics biodegradation and metabolism	29	181	181	1,084	0.56	0.79
Metabolism of cofactors and vitamins	17	131	181	1,084	0.86	0.92
Translation	7	129	181	1,084	1.00	1.00

*A represents the number of genes with non-synonymous SNPs involved in individual pathways; B represents the number of genes included in individual pathways; C represents total amount of genes involved in KEGG pathway among the genes with non-synonymous SNPs; D represents the total amount of genes involved in KEGG pathway in M. tb H37Rv genome; Q-values is the adjusted P-value, usually Q-values < 0.05 is defined as KEGG pathway significantly enriched*.

**Table 6:** The KEGG pathways enriched with non-synonymous SNPs affected genes in *M. bovis* AF2122_97 genome.

**Pathway**	**A**	**B**	**C**	**D**	***P*-value**	***Q*-value**
Carbohydrate metabolism	60	189	246	949	0.02	0.15
Transcription	3	5	246	949	0.02	0.15
Signal transduction	19	55	246	949	0.05	0.29
Biosynthesis of other secondary metabolites	12	36	246	949	0.11	0.39
Energy metabolism	42	144	246	949	0.14	0.39
Replication and repair	16	51	246	949	0.14	0.39
Membrane transport	16	52	246	949	0.16	0.39
Metabolism of other amino acids	24	82	246	949	0.19	0.41
Glycan biosynthesis and metabolism	6	20	246	949	0.24	0.46
Nucleotide metabolism	24	87	246	949	0.30	0.52
Metabolism of terpenoids and polyketides	30	115	246	949	0.43	0.67
Lipid metabolism	26	108	246	949	0.63	0.83
Folding, sorting and degradation	11	50	246	949	0.68	0.83
Amino acid metabolism	52	215	246	949	0.71	0.83
Xenobiotics biodegradation and metabolism	24	105	246	949	0.74	0.83
Metabolism of cofactors and vitamins	31	138	246	949	0.81	0.87
Translation	14	84	246	949	0.97	0.97

*A represents the number of genes with non-synonymous SNPs involved in individual pathways; B represents the number of genes included in individual pathways; C represents total amount of genes involved in KEGG pathway among the genes with non-synonymous SNPs; D represents the total amount of genes involved in KEGG pathway in M. bovis AF2122_97 genome; Q-values is the adjusted P-value, usually Q-values < 0.05 is defined as KEGG pathway significantly enriched*.

### Global comparison of the gene repertoires of *M. tb* strain 1458 and three other beijing family strains

A previous study determined that *M. tb* strain 1458, which is of bovine origin, belongs to the *M. tb* Beijing family genotype (Chen et al., 2009). Therefore, we compared the whole genome sequence of *M. tb* strain 1458 with those of three other Beijing family strains that were isolated from human TB patients, including two Chinese Beijing genotype strains [*M. tb* CCDC5079 (NC_021251) and CCDC5180 (NC_017522)] and one Indian strain [Beijing/NITR203 (NC_021054)]. Basically, *M. tb* strain 1458 is most closely related to the CCDC5079 strain, followed by the CCDC5180 strain (Figure 3). Coincidently, this variation of genetic distance agreed with the drug susceptibility phenotype, as the CCDC5079 strain is sensitive to all four first-line drugs, while the CCDC5180 strain is resistant to all four drugs (Zhang et al., 2011).

A pan-genome analysis and a Mauve alignment were used to compare the strains. The pan-genome analysis identified 3,373 orthologs as the core genome of the four genomes. These orthologs account for 74.6% of the 4,523 total genes of the four genomes (identified as the pan genome) (Table S5-1). In the group of 502 orthologs that are shared by any of the three genomes, 93 orthologs are shared by strains 1458, CCDC5180, and CCDC5079, and these include 15 PE/PPE family proteins and two multidrug resistance efflux proteins (BTB1458_1408 and BTB1458_1409) that were only absent in the *M. tb* Beijing/NITR203 genome (Table S5-2). Meanwhile, 395 orthologs are shared by the strain 1458, CCDC5079, and NITR203 genomes, and these include 50 antitoxins that are common to the three genomes, while only two antitoxins were found in the core genome (Tables S5-1, S5-2). The toxin-antitoxin (TA) system of these four genomes mainly consists of VapBC family proteins. VapC toxins, specifically their PilT N-terminus (PIN) domains, act as ribonucleases that cleave RNA molecules, thereby reducing the rate of translation (Arcus et al., 2011). Fifty-one toxins, including 28 VapC toxins, 15 putative toxins, two MazF toxins, and one ParE2 toxin were found in the core genome. It has been reported that TA systems are important for *M. tb* pathogenesis (Ramage et al., 2009); thus, the lack of antitoxins may indicate some loss of function in the CCDC5079 and CCDC5180 genomes. A total of 201 orthologs that are shared by any two genomes were identified (Table S5-3). Most of these orthologs are hypothetical proteins of unknown function.

Unique genes were also identified in each genome (Table S5-4). For the strain 1458, 33 genes are considered to be unique, including 30 hypothetical protein-encoding genes, one PE-PGRS family protein-encoding gene, and one putative collagen-like protein-encoding gene. For *M. tb* strain CCDC5079, 70 genes are considered to be unique, including 57 hypothetical protein-encoding genes, two lipoprotein-encoding genes, one PE-PGRS family protein-encoding gene, one transposase-encoding gene, and one putative collagen-like protein-encoding gene. For the CCDC5180 genome, 43 genes are considered to be unique, including 22 hypothetical protein-encoding genes, 10 PE/PPE family protein-encoding genes, and two MCE family protein-encoding genes. For the NITR203 genome, 183 genes are considered to be unique, including 110 hypothetical protein-encoding genes, 18 PE/PPE family protein-encoding genes, nine transposase-encoding genes, eight phiRv1 phage-related protein-encoding genes, one phospholipase C4 (MTP40 antigen)-encoding gene, one putative membrane-associated phospholipase C2-encoding gene, one polyketide synthase (Pks13)-encoding gene, one putative ESAT-6-like protein-encoding gene, one putative toxin (VapC16)-encoding gene, and one antitoxin-encoding gene.

In summary, the unique genes of the four genomes totaled 329, and among them, the most common category is that of hypothetical genes, comprising 66.5% of the unique genes, followed by PE/PPE family protein-encoding genes, which comprise 9.1% of the unique genes (Table S5-4). For the individual genomes, strain 1458 has the fewest unique genes (33), 91% (30) of which are hypothetical genes, and 3% (1) of which are PE/PPE family protein-encoding genes, while strain NITR203 has the most unique genes (183), mainly resulting from three inserted fragments (described below), 60.1% (110) of which are hypothetical genes, and 9.8% (18) of which are PE/PPE family protein-encoding genes.

Through the Mauve alignment, three unique insertion fragments were identified in the NITR203 genome (Figure S2). The first (nt 79575 to 83038) affects four genes, including those encoding RNA-directed DNA polymerase, a hypothetical protein, a glutamine-transport transmembrane protein ATP-binding cassette transporter, and a glutamine-transport ATP-binding cassette transporter (nt 479312910 to 479312913). The second (nt 1779126 to 1788367) affects 13 genes that consist of a phiRv1 phage-related gene cluster (nt 479314486 to 479314498). The third (nt 1988260 to 1998428) affects nine genes, including those encoding three transposases, one cutinase, Cut1, one PE-PGRS family protein, one acyltransferase, and three hypothetical proteins (nt 479314672 to 479314680). These genes were also identified as unique in the pan-genome analysis (Table S5-4).

### Mutation-affected genes in *M. tb* strain 1458 and three other beijing genotype strains

Comparison of the *M. tb* 1458 genome with three Beijing genomes (CCDC5079, CCDC5180, and NITR203) of human origin revealed that 315 SNPs are specific to CCDC5079, 1,005 SNPs are specific to CCDC5180, and 2,762 SNPs are specific to NITR, while 133 SNPs are unique to the strain 1458 (Table S6). These unique SNPs of the strain 1458 caused non-synonymous variations in 70 genes. A KEGG pathway enrichment analysis only revealed enrichment in RNA polymerase (*p* < 0.01). Moreover, 127 of the 133 unique SNPs in strain 1458 are either different to those in the *M. bovis* genome, implying that strain 1458 might have evolved a virulence mechanism that differs from those of *M. bovis* or the other *M. tb* Beijing family strains of human origin.

### Comparison of the IS*6110* distribution between the strain 1458 and two beijing genotype strains

As described above, *M. tb* strain 1458 has 21 copies of IS*6110*, 11 of which are distributed within ORFs, while 10 lie between ORFs. Thus, we analyzed the distribution of IS*6110* in two other Chinese Beijing genotype strains of human origin, excluding the Indian strain NITR203. The results showed that 18 and 21 copies of IS*6110* are located in the CCDC5180 and CCDC5079 genomes, respectively (Table S7).

Compared with *M. tb* strain 1458, the strain CCDC5079 has two special insertions in a cation efflux system protein-encoding gene and *hsdM2*, in addition to the copies that are present in hypothetical protein-, PE/PPE family protein-, and transposase-encoding genes. However, neither strain was shown to contain an insertion at the upstream of *phoP* and *esxS* genes, suggesting that the virulence of strain 1458 may differ from that of the other two strains.

### Indel analysis of *M. tb* strain 1458 and two other beijing family strains

We analyzed the indels between *M. tb* strain 1458 and two other Chinese Beijing genotype strains, and the unique insertions of each strain are shown in Table S8. There are 54 unique insertion fragments in these three strains, and they are mainly enriched in genes encoding PE/PPE family proteins (16/54, 29.6%), transposases (17/54, 31.5%), and hypothetical proteins (9/54, 16.6%). For the strain 1458, 15 unique insertion fragments were identified. Of these, a 292 bp insertion in the 5′ terminus of *vapB16* is considered to be unique. The *vapB16* is a member of the TA loci, and this locus is conserved in other pathogenic strains of the MTBC, including *M. bovis* and *M. avium*. For strain CCDC5180, 22 unique insertion fragments were identified. Of these, two insertion fragments, 288 and 302 bp, respectively, were identified in protoheme IX farnesyltransferase. For strain CCDC5079, 17 unique insertion fragments were identified, and of these, a 1,731 bp insertion fragment, involving 542 bp at the 3′ terminus of a gene encoding a member of the phthalate permease family, was unique.

## Discussion

Although TB in humans and other species caused by the MTBC is considered to be a zoonotic infectious disease, MTBC members have apparent host preferences, as evidenced by the fact that *M. tb* causes human TB, while *M. bovis* is responsible for TB in bovines and a wide range of other animals. Occasionally, a cross-species infection occurs when the bacilli infect another species. For example, humans can become infected by *M. bovis* by drinking unpasteurized contaminated milk or inhaling contaminated aerosols generated by cattle with bovine TB (Perez-Lago et al., 2014). However, the host specificity varies greatly among MTBC members; for example, *M. tb* mainly causes human TB, while *M. bovis* has a wide host range.

For over 100 years, *M. tb* has been considered to be avirulent to cattle (Whelan et al., 2010). However, it is difficult to explain the fact that *M. tb* strains were the only strains isolated from lesioned lung tissues or lymph nodes of some cattle with TB (Prasad et al., 2005; Chen et al., 2009; Fetene et al., 2011). To address this issue, we chose one well characterized strain, *M. tb* 1458, of bovine origin to annotate its genome. The purpose was to determine whether it has obtained some special genetic properties contributing to its ability to successfully infect cattle. Strain 1458 is a *M. tb* strain belonging to the Beijing family, and it was previously isolated from lesioned lung tissue. An experimental infection demonstrated that it can infect cattle and cause metabolomics disorders and immunological responses that are similar to those caused by *M. bovis* (Chen et al., 2009, 2013).

In this study, the phylogenetic analysis confirmed that strain 1458 is most closely related to the Chinese *M. tb* Beijing family strain CCDC5079, which is of human origin. The global comparison of the gene repertoires of *M. tb* strain 1458 and three other Beijing family strains showed that *M. tb* strain 1458 has the fewest unique genes. Therefore, *M. tb* strain 1458 is just a typical strain of *M. tb* Beijing family. However, each strain indeed exhibited some specific modifications which might be associated with the host adaptation. When the unique genes of the four Beijing family strains were analyzed, over half of them were hypothetical genes, and most of the remaining unique genes encoded PE/PPE family proteins, which play an important role in antigenic variation (Soto et al., 2004). In addition, although most of the IS*6110*- and SNP-affected genes are hypothetical, the few interrupted genes might affect the host adaptation. For example, only strain 1458 has the IS6110 insertion in the upstream region of the *phoP* and *esxS* genes. A previous study showed that *phoP* transcription was strongly upregulated when the promoter region contained an IS6110 element (Gonzalo-Asensio et al., 2014). We confirmed the transcription difference in the four genes *esxS, phoP, dnaN*, and *ctpD* between *M. tb* 1458 and H37Rv and *M. bovis*, but if this difference was caused by IS*6110* upstream insertion and if this insertion led to the host adaptation of *M. tb* 1458 in cattle remains to be further investigated. In addition this insertion in these critical genes' upstream might modify the virulence and immunogenicity, because these genes are known to be responsible for *M. tb* virulence (Soto et al., 2004).

In conclusion, the genome of *M. tb* strain 1458, which is of bovine origin, was sequenced, and an extensive comparative analysis revealed that although this strain did not experience significant mutations, IS*6110* and other IS insertions and non-synonymous SNPs and indel in critical genes, such as *phoP, esxS*, and PE/PPE family protein-encoding genes, might contribute to its bovine adaptation and modify its virulence and immunogenicity in cattle.

## Author contributions

AG and HZ: Conceived and designed the experiments, and revised the paper; XX, DD, RW, HL, JW, XJZ, and XFZ: Performed the experiments; YC and TW: Analyzed the data; HC, XL, and YZ: Contributed reagents/materials/analysis tools; XX, DD, and RW: Wrote the draft.

### Conflict of interest statement

The authors declare that the research was conducted in the absence of any commercial or financial relationships that could be construed as a potential conflict of interest.

## Supplementary material

Figure S1 Genome structure comparison of *M. tb* 1458, H37Rv, and *M. bovis* AF2122_97. The graph represents an alignment of the colinear blocks, identified by MAUVE, that are conserved in three closely related genomes: *M. tb* 1458, H37Rv, and *M. bovis* AF2122_97. Only one red block and little blank spaces are found in these three genomes, indicating their highly similarity in genome structure.

Figure S2 Genome structure comparison of *M. tb* 1458 and three other Beijing family strains. The graph represents an alignment of the colinear blocks, identified by MAUVE, that are conserved in four evolutionarily close genomes: *M. tb* 1458, CCDC5079 (NC_021251), CCDC5180 (NC_017522), and Beijing/NITR203 (NC_021054). Three obvious blank regions are identified only in Beijing/NITR203 (NC_021054) genome, which are considered to be unique fragments.

Table S1 COG annotation of *M. tb* 1458.

Table S2 IS elements distribution in *M. tb* 1458.

Table S3 Pan-genome analysis results of *M. tb* 1458, H37Rv and *M. bovis* AF2122_97.

Table S3-1 Core genome of *M. tb* 1458, H37Rv and *M. bovis* AF2122_97 genomes.

Table S3-2 Specific genes of *M. tb* 1458 genome.

Table S3-3 Specific genes of H37Rv genome.

Table S3-4 Specific genes of *M. bovis* AF2122_97 genome.

Table S3-5 Common genes of *M. tb* 1458 and *M. bovis* AF2122_97 genomes.

Table S3-6 Common genes of *M. tb* 1458 and H37Rv genomes.

Table S3-7 Common genes of H37Rv and *M. bovis* AF2122_97 genomes.

Table S4 SNPs between *M. tb* 1458, H37Rv and *M. bovis* AF2122_97.

Table S4-1 SNPs of *M. tb* 1458 based on H37Rv.

Table S4-2 SNPs of *M. tb* 1458 based on *M. bovis* AF2122_97.

Table S5 Pan-genome analysis results of *M. tb* 1458 and three other Beijing family strains.

Table S5-1 Core genome of *M. tb* 1458 and three other Beijing family strains.

Table S5-2 Orthologs shared by any three genomes.

Table S5-3 Orthologs shared by any two genomes.

Table S5-4 Unique genes of the four genomes.

Table S6 Common SNPs in *M. tb* 1458 compared to three other Beijing genomes.

Table S7 IS6110 distribution of CCDC5079 (NC_021251) and CCDC5180 727 (NC_017522).

Table S7-1 IS6110 distribution of CCDC5079 (NC_021251).

Table S7-2 IS6110 distribution of CCDC5180 (NC_017522).

Table S8 Unique insertion between *M. tb* 1458 and two other Beijing genotype strains.
