# Supplementary figures and images for: Comparative Genomics of a Bovine Mycobacterium tuberculosis Isolate and Other Strains Reveals Its Potential Mechanism of Bovine Adaptation
Source: Front Microbiol. 2017 Dec 12;8:2500. doi: 10.3389/fmicb.2017.02500 (PMC5733104; doi:10.3389/fmicb.2017.02500)

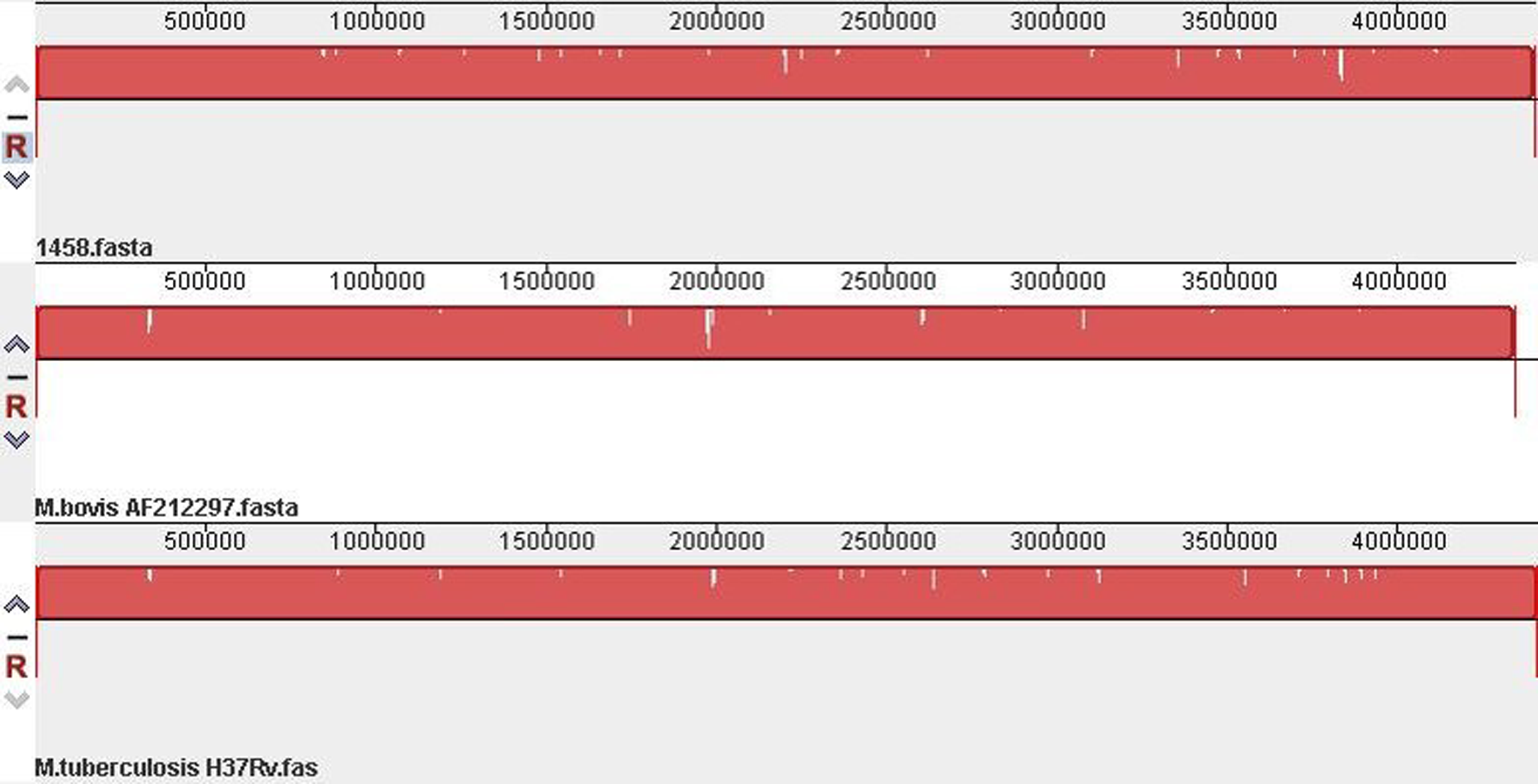

Supplement: Figure S1 — Genome structure comparison of M. tb 1458, H37Rv, and M. bovis AF2122_97. The graph represents an alignment of the colinear blocks, identified by MAUVE, that are conserved in three closely related genomes: M. tb 1458, H37Rv, and M. bovis AF2122_97. Only one red block and little blank spaces are found in these three genomes, indicating their highly similarity in genome structure. [file Image1.TIF]

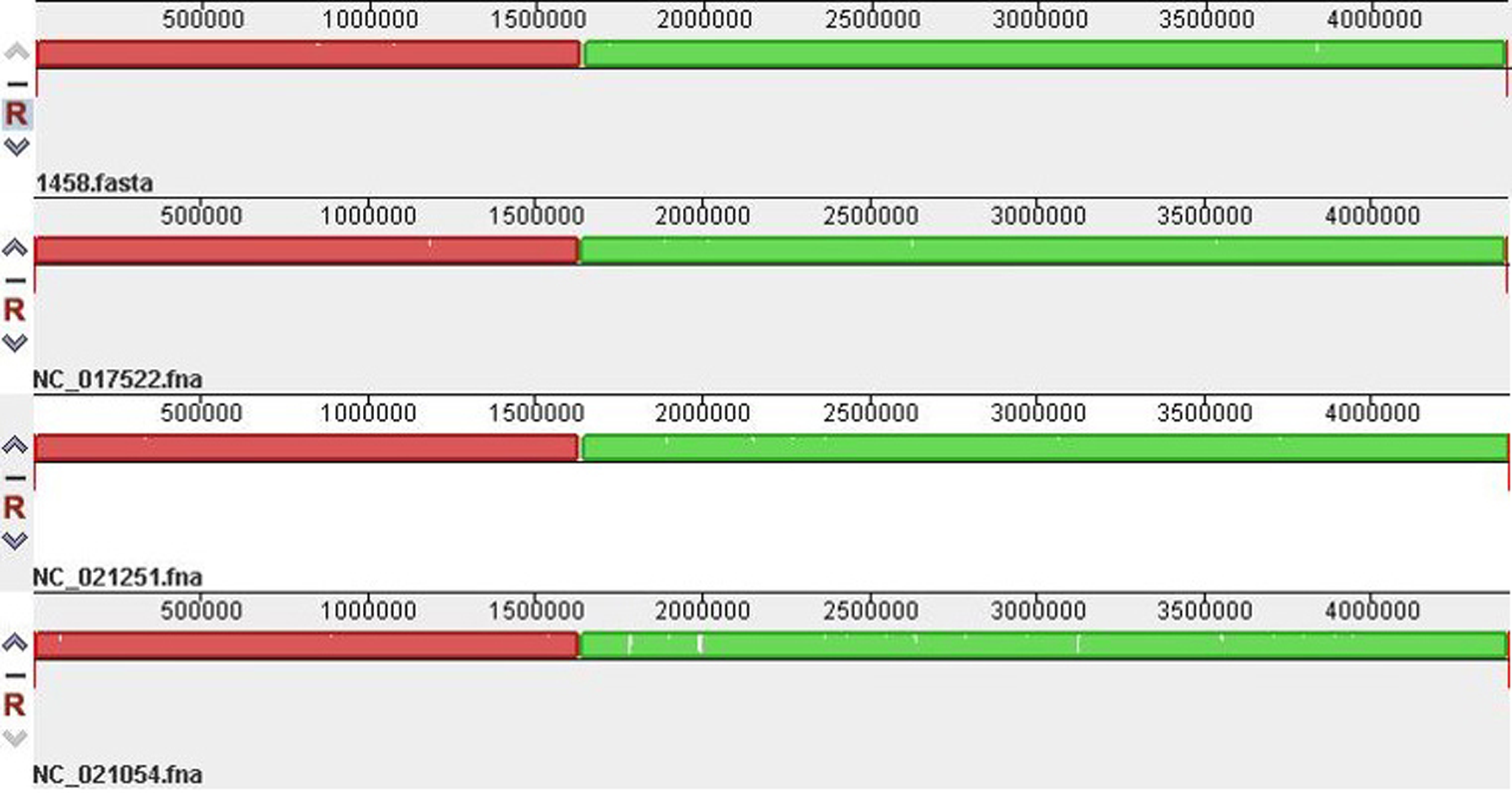

Supplement: Figure S2 — Genome structure comparison of M. tb 1458 and three other Beijing family strains. The graph represents an alignment of the colinear blocks, identified by MAUVE, that are conserved in four evolutionarily close genomes: M. tb 1458, CCDC5079 (NC_021251), CCDC5180 (NC_017522), and Beijing/NITR203 (NC_021054). Three obvious blank regions are identified only in Beijing/NITR203 (NC_021054) genome, which are considered to be unique fragments. [file Image2.TIF]
